# Negative Elongation Factor (NELF) Inhibits Premature Granulocytic Development in Zebrafish

**DOI:** 10.3390/ijms23073833

**Published:** 2022-03-30

**Authors:** Mengling Huang, Abrar Ahmed, Wei Wang, Xue Wang, Cui Ma, Haowei Jiang, Wei Li, Lili Jing

**Affiliations:** 1 Engineering Research Center of Cell & Therapeutic Antibody, Ministry of Education, School of Pharmacy, Shanghai Jiao Tong University, Shanghai 200240, China; morninghuang@sjtu.edu.cn (M.H.); abrar22@sjtu.edu.cn (A.A.); vivid.sjtu.edu.cn@sjtu.edu.cn (W.W.); anewang@sjtu.edu.cn (X.W.); ma-cui@sjtu.edu.cn (C.M.); haoweijiang@sjtu.edu.cn (H.J.); 2 Core Facility and Technical Service Center, School of Pharmacy, Shanghai Jiao Tong University, Shanghai 200240, China; liweishen@sjtu.edu.cn

**Keywords:** zebrafish, negative elongation factor, RNA polymerase II promoter proximal pausing, granulocytic development

## Abstract

Gene expression is tightly regulated during hematopoiesis. Recent studies have suggested that RNA polymerase II (Pol II) promoter proximal pausing, a temporary stalling downstream of the promoter region after initiation, plays a critical role in regulating the expression of various genes in metazoans. However, the function of proximal pausing in hematopoietic gene regulation remains largely unknown. The negative elongation factor (NELF) complex is a key factor important for this proximal pausing. Previous studies have suggested that NELF regulates granulocytic differentiation in vitro, but its in vivo function during hematopoiesis remains uncharacterized. Here, we generated the zebrafish mutant for one NELF complex subunit Nelfb using the CRISPR-Cas9 technology. We found that the loss of *nelfb* selectively induced excessive granulocytic development during primitive and definitive hematopoiesis. The loss of *nelfb* reduced hematopoietic progenitor cell formation and did not affect erythroid development. Moreover, the accelerated granulocytic differentiation and reduced progenitor cell development could be reversed by inhibiting Pol II elongation. Further experiments demonstrated that the other NELF complex subunits (Nelfa and Nelfe) played similar roles in controlling granulocytic development. Together, our studies suggested that NELF is critical in controlling the proper granulocytic development in vivo, and that promoter proximal pausing might help maintain the undifferentiated state of hematopoietic progenitor cells.

## 1. Introduction

Hematopoiesis is a complex process involving the collaboration of intrinsic hematopoietic transcriptional regulators and signaling molecules from the microenvironment [1]. Defects in the formation and differentiation of hematopoietic cells often result in various diseases such as anemia and leukemia.

To ensure that blood cells are properly formed, the expression of key regulatory genes in hematopoiesis is strictly regulated at the transcriptional level. Transcription does not function as a simple ‘on-off’ switch, but instead involves various rate-limiting steps that each contribute to the overall transcription rate and efficiency [2,3,4,5]. Increasing studies revealed that transcriptionally engaged RNA polymerase II (Pol II) on many metazoan genes experiences a temporary stalling at 20 to 120 nucleotides downstream of the promoter region after initiation, which is described as the promoter proximal pausing of Pol II [6]. This pausing is widespread in the expressed genome, ranging from 30% to 70% detection among different studies, indicating its critical role in gene regulation [5,7,8,9].

Promoter proximal pausing stabilization requires two important protein complexes: the pausing negative elongation factor (NELF) and DEB-sensitive inducing factor (DSIF) [10,11]. The NELF complex contains four subunits (A, B, C/D and E) [11,12]. Accumulating evidence has suggested an important role of NELFB in gene expression, and NELFB expression is strictly regulated during development [13,14,15,16,17]. The release of paused Pol II into productive elongation requires the activity of positive transcription elongation factor b (P-TEFb), which phosphorylates Pol II, DSIF, and NELF [6,18]. NELF dissociates from chromatin upon its phosphorylation, whereas DSIF switches to a positive elongation factor [19,20,21]. Despite extensive biochemical studies, limited research exists regarding the function of these complexes in vivo. A recent study using in vitro hematopoietic cell culture suggested that NELF plays negative roles in granulocytic differentiation [17], but, its in vivo functions in other hematopoietic lineage differentiation remain unknown.

As a powerful genetic model, zebrafish have increasingly been used to study hematopoietic development, with many advantages. They provide a convenient and fast tool to study gene regulation and functions in vivo [1]. Although hematopoiesis in zebrafish occurs in a spatially unique manner compared to other vertebrates, the developmental processes and genetics programs are still highly conserved [1]. Similar to the mammalians, hematopoiesis in zebrafish also involves a primitive wave and definitive wave [22]. Starting at approximately 12 h post-fertilization (hpf), primitive hematopoietic cells are born in the anterior lateral plate mesoderm (ALPM) and the intermediate cell mass (ICM) [1,23]. During this wave, the cells in the ALPM generate mainly myeloid cells, including macrophages and neutrophils, while the cells in ICM (also known as the posterior lateral plate mesoderm) mainly give rise to erythrocytes and potentially neutrophils [24,25,26,27]. The definitive wave of hematopoiesis initiates at around 30 hpf [22]. The definitive hematopoietic stem cells (HSCs) emerge from the aorta-gonad-mesonephros (AGM) region, and these HSCs migrate to the caudal hematopoietic tissue (CHT) in the tail, finally residing in the kidney marrow (equivalent to mammalian bone marrow) [22]. Different from primitive hematopoiesis, the definitive wave generates all lineage blood cells, including myeloid cells and lymphoid cells [22].

In this study, we used zebrafish as a model system to investigate the role of Nelfb and other NELF subunits during hematopoietic development. Our results demonstrated that Nelfb specifically controls primitive and definitive granulocytic development at the appropriate time by regulating the expression of lineage genes through Pol II pausing. We further demonstrated that other NELF subunits (Nelfa and Nelfe) play similar roles in granulopoiesis. Together, these results indicate that NELF plays an important role in preventing premature granulopoiesis.

## 2. Results

### 2.1. Expression of nelfb during Development and Generation of Zebrafish nelfb Mutants

**Figure 1:**
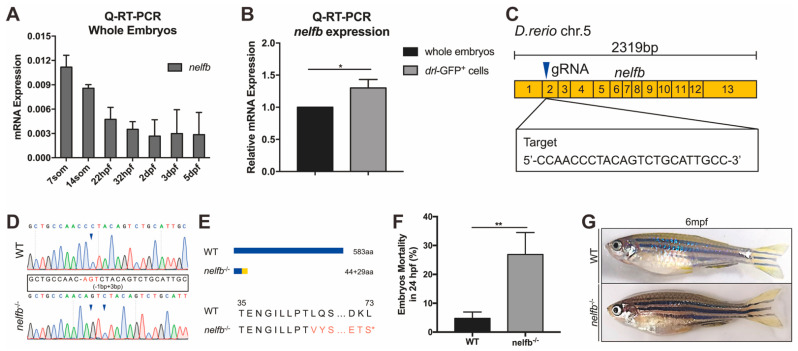
Expression of *nelfb* during development and generation of zebrafish *nelfb* mutants. (**A**) Q-RT-PCR analysis of whole embryos to show the expression of *nelfb* at different stages. The relative expression of *nelfb* is normalized to the expression of *β-actin*. (**B**) Q-RT-PCR analysis of the relative expression of *nelfb* in sorted *drl*-GFP^+^ cells compared with the whole embryo lysates at 24 hpf. (**C**) The schematic diagram of *nelfb* cDNA and the targeted region of guide RNA. The target DNA sequence is shown in the rectangle. (**D**) Sanger sequencing analysis of PCR fragments containing the gRNA targeted region from WT and *nelfb* deficient homozygotes. The deleted nucleotides are replaced by -, and the inserted nucleotides are in red, as shown in the rectangle. (**E**) Schematic representation and amino acid sequences of the wild type Nelfb and the predicted truncated protein. (**F**) The mortality rate of embryos at 24 hpf (*n* = 100–300 embryos per group). (**G**) Images of adult zebrafish at 6 mpf. All results are presented as the mean ± SD from three independent experiments (*t* test, * for *p* < 0.05, ** for *p* < 0.01). WT, wildtype; hpf, hours post-fertilization; mpf, months post-fertilization.

NELF is composed of four subunits (NELFA, B, C/D, and E) that are interdependent [28]. One of the most extensively studied subunits is NELFB. To investigate the roles of Nelfb during embryonic development, we first examined the expression of *nelfb* at different development stages using quantitative reverse transcription PCR (q-RT-PCR). *nelfb* was abundantly expressed at early developmental stages, but decreased when embryos proceeded to later stages (Figure 1A). To analyze the expression of *nelfb* in blood cells, we purified *draculin* (*drl*)^+^-hematopoietic cells from Tg(*drl:GFP*) fish by fluorescence-activated cell sorting (FACS). The *draculin* element is active during early development in all lineages derived from the anterior and posterior hematopoietic population; thus; Tg(*drl:GFP*) covers all hematopoietic cells [29,30]. Compared with the whole embryo lysates, *nelfb* was more abundantly expressed in hematopoietic cells (Figure 1B), supporting its potential function in hematopoiesis.

We next generated a *nelfb* mutant using the CRISPR/Cas9 system (Figure 1C). The 583 aa protein Nelfb is encoded by the 2319 bp gene *nelfb*, which contains 13 exons. A guide RNA that targets the second exon was designed and co-injected with Cas9 mRNA into one-cell stage wildtype (WT) embryos. After screening several founders that transmitted to F1 progeny, a stable *nelfb*^−/−^ line was established that has a frameshift mutation and a premature stop codon (Figure 1D,E). The altered transcript is predicted to encode a truncated Nelfb protein with 73 amino acids (Figure 1E).

The mortality rates in *nelfb*^−/−^ embryos were significantly increased compared to the WT at 24 hpf (Figure 1F), supporting that Nelfb plays important roles in zebrafish embryogenesis. The embryos that survived developed into adults (Figure 1G). The adult *nelfb*^−/−^ mutants did not show obvious defects, but they were more susceptible to infections than their WT siblings (data not shown).

### 2.2. nelfb Deficiency Leads to Accelerated Granulocytic Development during Primitive Hematopoiesis

Zebrafish hematopoiesis, which mainly proceeds through two successive waves (primitive wave and definitive wave), is highly conserved when compared to that of mammals [1]. We first examined the function of Nelfb in primitive hematopoietic development. To do this, whole-mount in situ hybridization (WISH) was performed at different stages to detect primitive hematopoiesis markers.

**Figure 2:**
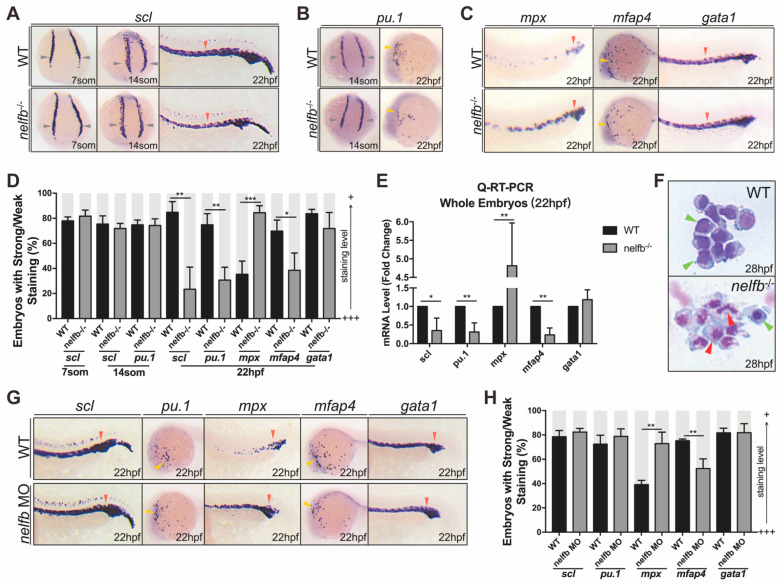
Nelfb deficiency leads to accelerated granulocytic development during primitive hematopoiesis. (**A**) WISH for *scl* in WT and *nelfb*^−/−^ embryos at 7-, 14-somite stages and 22 hpf. (**B**) WISH for *pu.1* in WT and *nelfb*^−/−^ embryos at 14-somite stage and 22 hpf. (**C**) WISH for *mpx*, *mfap4,* and *gata1* in WT and *nelfb*^−/−^ embryos at 22 hpf. (**D**) Quantification of WISH results in (**A**–**C**) (*n* = 40–60 embryos per group). (**E**) Q-RT-PCR analysis of gene expression in *nelfb*^−/−^ and WT embryos at 22 hpf. Gene expression is normalized to *β*-actin and presented as fold-change relative to WT. (**F**) May–Grünwald–Giemsa staining of peripheral blood in WT and *nelfb*^−/−^ embryos at 28 hpf. Green arrowheads indicate precursors; red arrowheads indicate granulocytes. (**G**) WISH for *scl*, *pu.1*, *mpx*, *mfap4*, and *gata1* in WT and *nelfb* morphants at 22 hpf. (**H**) Quantification of WISH results in (**G**) (*n* = 20–40 embryos per group). All results are presented as the mean ± SD from three independent experiments (*t* test, * for *p* < 0.05, ** for *p* < 0.01, *** for *p* < 0.001). Grey arrowheads, yellow arrowheads, and red arrowheads in (**A**–**C**,**G**), respectively, indicate ICM, ALPM, and PLPM. “+++” and “+” in (**D**,**H**) respectively represent strong staining and weak staining. WISH, whole-mount in situ hybridization; ICM, intermediate cell mass; ALPM, anterior lateral plate mesoderm; PLPM, posterior lateral plate mesoderm.

The loss of *nelfb* had no apparent effects on hematopoietic progenitor cell development at 7- and 14-somite stages, as indicated by normal *scl* expression (Figure 2A,D). However, *scl* expression was dramatically reduced at later stages (22 hpf) (Figure 2A,D). Similarly, the expression of *pu.1* in *nelfb*^−/−^ mutants at the 14-somite stage was comparable to WT embryos, with a decrease at 22 hpf, suggesting an abnormal myeloid progenitor development (Figure 2B,D). These data indicated that, although primitive hematopoiesis before the 14-somite stage is not affected by *nelfb* knockout, defects in primitive hematopoietic progenitors, such as the myeloid progenitors, initiates from the later stage, around 22 hpf.

At 22 hpf, *nelfb* knockout also resulted in a slight loss of the macrophages expressing marker *mfap4*, but it led to a striking increase in the expression of neutrophil marker *mpx*, which encodes a proinflammatory enzyme myeloperoxidase mainly expressed in neutrophils [31]. Meanwhile, erythroid development appeared to be relatively normal in the *nelfb*^−/−^ mutant, as comparable *gata1* mRNA expression was observed (Figure 2C,D). These results were also verified by q-RT-PCR (Figure 2E). Moreover, no significant change of blood cell proliferation or apoptosis, but a slight inflammatory activation, was observed (Supplementary Figure S1). Together, these results suggested that *nelfb* depletion selectively causes premature granulocytic development at the cost of hematopoietic progenitor cells during primitive hematopoiesis.

Differentiated neutrophils are characterized by banded and segmented nuclei [32]. Therefore, blood cells were collected at 28 hpf to stain nuclei with May–Grünwald–Giemsa stain (Figure 2F). The results showed a significant increase in differentiated granulocytic cells in the *nelfb*^−/−^ mutants compared with WT at 28 hpf, supporting that primitive neutrophil development is indeed accelerated by *nelfb* mutation.

To verify the results obtained from the *nelfb*^−/−^ mutants, we used a previously published morpholino [33] to knock down *nelfb* and examined primitive hematopoiesis (Supplementary Figure S2A,B). At 22 hpf, although the *nelfb* morphants showed similar levels of *scl* and *pu.1* expression with WT, the *mpx* stained neutrophils were significantly increased in morphants, with decreased macrophages marked by *mfap4* (Figure 2G). Meanwhile, primitive erythroid development was normal (Figure 2G). Due to the potential for limited efficiency of morpholino-mediated knockdown of *nelfb*, these results also supported that *nelfb* deficiency accelerates granulocytic differentiation.

### 2.3. Inhibition of Pol II Elongation Rescues Primitive Hematopoiesis in nelfb^−/−^ Embryos

**Figure 3:**
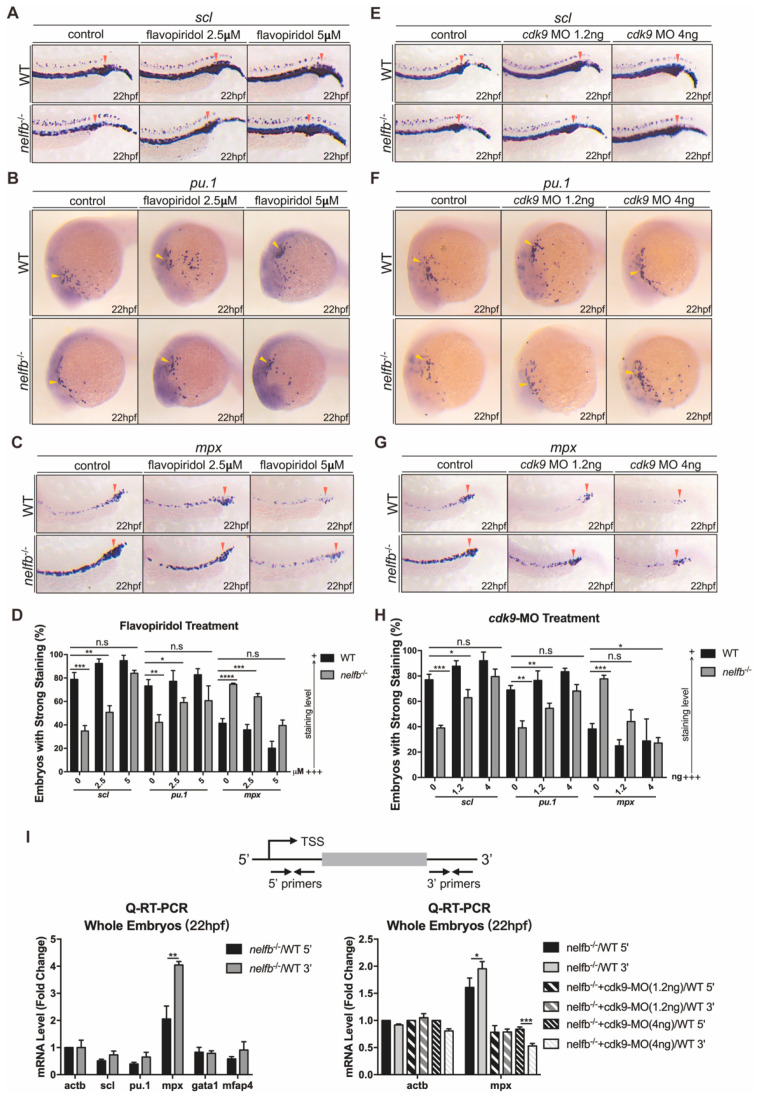
Inhibition of Pol II elongation rescues primitive hematopoiesis in *nelfb*^−/−^ embryos. (**A**–**C**) WISH for *scl* (**A**), *pu.1* (**B**) or *mpx* (**C**) at 22 hpf in WT and *nelfb*^−/−^ embryos treated with DMSO, 2.5 µM flavopiridol, or 5 µM flavopiridol. (**D**) Quantification of WISH results in (**A**–**C**) (*n* = 30–50 embryos per group). (**E**–**G**) WISH for *scl* (**E**)*, pu.1* (**F**)*,* or *mpx* (**G**) at 22 hpf in WT and *nelfb*^−/−^ embryos without or with *cdk9* MO injection (1.2 ng or 4 ng). (**H**) Quantification of WISH results in (**E**–**G**) (*n* = 30–50 embryos per group). (**I**) Upper panel shows the position of primers used in Q-RT-PCR analysis. Primers for the 5′ transcripts are located within 120 bp from transcription start site (TSS), and primers for the 3′ transcripts are in the 3′ coding region or 3′UTR. Lower-left panel shows Q-RT-PCR analysis of 5′ and 3′ transcripts of hematopoiesis-related genes in WT and *nelfb*^−/−^ embryos at 22 hpf. Lower-right panel shows Q-RT-PCR analysis of 5′ and 3′ transcripts of *mpx* gene in 22 hpf *nelfb*^−/−^ embryos without or with *cdk9* MO injection (1.2 ng or 4 ng). Gene expression is normalized to the 5′ transcript of *β*-actin and shown as fold-change relative to WT, following the methods in the previous study [35]. All results are presented as the mean ± SD from three independent experiments (*t* test, * for *p* < 0.05, ** for *p* < 0.01, *** for *p* < 0.001, **** for *p* < 0.0001). Yellow arrowheads and red arrowheads in (**A**–**C**) and (**E**–**G**), respectively, indicate ALPM and PLPM. “+++” and “+” in (**D**,**H**) respectively represent strong staining and weak staining. MO, morpholino.

Losing Pol II pausing may lead to premature release of Pol II into the elongation phase. As one of the subunits of P-TEFb, CDK9 is critical for promoting Pol II into productive elongation [34]. To examine if the defects in *nelfb*^−/−^ were caused by premature Pol II elongation, WT and *nelfb*^−/−^ embryos were treated with CDK9 inhibitor flavopiridol from the 2-somite stage to 22 hpf. The results showed that progenitor cells and myeloid precursors were greatly increased, while neutrophils were notably decreased, in *nelfb*^−/−^ embryos by treatment with flavopiridol at the concertation of 2.5–5 µM (Figure 3A–D). The progenitor cells and myeloid precursors were also increased, and neutrophils were decreased in WT embryos with the same flavopiridol treatment, but to a much lower degree. In addition, we used a splicing-blocking morpholino to knock down Cdk9 in the embryos [33]. Both low- and high-doses of morpholino reversed the phenotypes in *nelfb*^−/−^ embryos, and both showed mild similar effects in WT embryos, consistent with the phenotypes in the flavopiridol treatment (Figure 3E–H). Collectively, these results suggested that primitive progenitor formation and myeloid development require a tightly controlled release of paused Pol II. Losing pausing factors like Nelfb leads to premature granulocytic differentiation, which can be reversed by inhibiting Pol II elongation.

To further test if the loss of *nelfb* indeed affects the transcription elongation of hematopoietic genes, we assayed blood gene transcripts near the 5′ or 3′ end using q-RT-PCR to study Pol II position (Figure 3I), as suggested in the previous study [35]. The expression of 5′ ends and 3′ ends of *scl*, *pu.1, gata1*, and *mfap4* gene transcripts were all slightly decreased or unaffected, and there was a slightly greater increase in transcription products in the 3′ ends than in the 5′ ends for these genes (Figure 3I). However, we detected a strongly increased expression of the 5′ ends of *mpx* gene transcripts in *nelfb*^−/−^. Moreover, the 3′ ends of *mpx* gene transcripts in *nelfb*^−/−^ showed a dramatical increase, which was reduced by *cdk9* morpholino (Figure 3I). These results suggested that the loss of Pol II pausing leads to an increased Pol II elongation, particularly at the loci of the neutrophil granulocyte specific gene *mpx*, as well as a subsequent increased expression, and that the increased *mpx* expression can be reversed by inhibiting the elongation.

### 2.4. Expression of Human NELFB in Hematopoietic Cells Partially Rescues Granulopoiesis in nelfb^−/−^ Embryos

**Figure 4:**
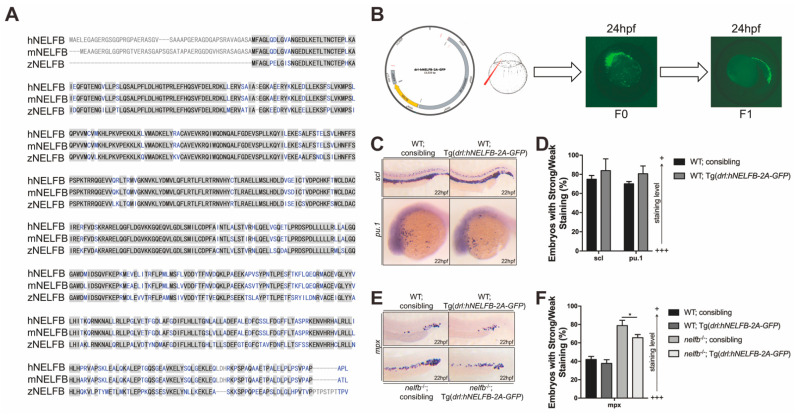
Expression of human *NELFB* in hematopoietic cells partially rescues granulopoiesis in *nelfb*^−/−^ embryos. (**A**) Alignment of human, mouse, and zebrafish NELFB proteins. Identical and similar amino acids are shaded in gray. (**B**) Generation of Tg(*drl:hNELFB-2A-GFP*) fish and GFP expression. (**C**) WISH for *scl* or *pu.1* in WT and Tg(*drl:hNELFB-2A-GFP*) embryos at 22 hpf. (**D**) Quantification of WISH results in (**C**) (*n* = 40–60 embryos per group). (**E**) WISH for *mpx* in WT, Tg(*drl:hNELFB-2A-GFP*), *nelfb*^−/−^ and *nelfb*^−/−^; Tg(*drl:hNELFB-2A-GFP*) embryos at 22 hpf. (**F**) Quantification of WISH results in (**E**) (*n* = 20–30 embryos per group). All results are presented as the mean ± SD from three independent experiments (*t* test, * for *p* < 0.05). “+++” and “+” in (**D**,**F**) respectively represent strong staining and weak staining. *hNELFB*, *human NELFB*.

Since it is possible that the loss of Nelfb in adjacent tissues affects hematopoietic development in *nelfb*^−/−^, we next studied if Nelfb functions directly in hematopoietic cells to regulate primitive granulopoiesis. The zebrafish Nelfb protein displays a 76.8% identity to the human NELFB protein and a 76.9% identity to the mouse NELFB protein, indicating that NELFB is highly evolutionarily conserved in vertebrates (Figure 4A). To investigate the cell autonomous regulation of Nelfb in blood, we constructed a stable transgenic line with the plasmid containing the human *NELB* (*hNELFB*) gene under the control of the *draculin* promoter [30], Tg (*drl-hNELFB-2A-GFP*) to overexpress *hNELFB* in blood cells (Figure 4B).

Tg(*drl:hNELFB-2A-GFP*) overexpression in WT embryos only led to a mild increase in stem cells and myeloid progenitors and did not affect neutrophil development (Figure 4C–F). However, Tg(*drl:hNELFB-2A-GFP*) overexpression in *nelfb*^−/−^ partially inhibited the premature granulocytic development, supporting the idea that *NELFB* likely functions in blood cells to control granulopoiesis (Figure 4E,F).

### 2.5. Other NELF Subunits Play Similar Roles in Primitive Granulocytic Development

**Figure 5:**
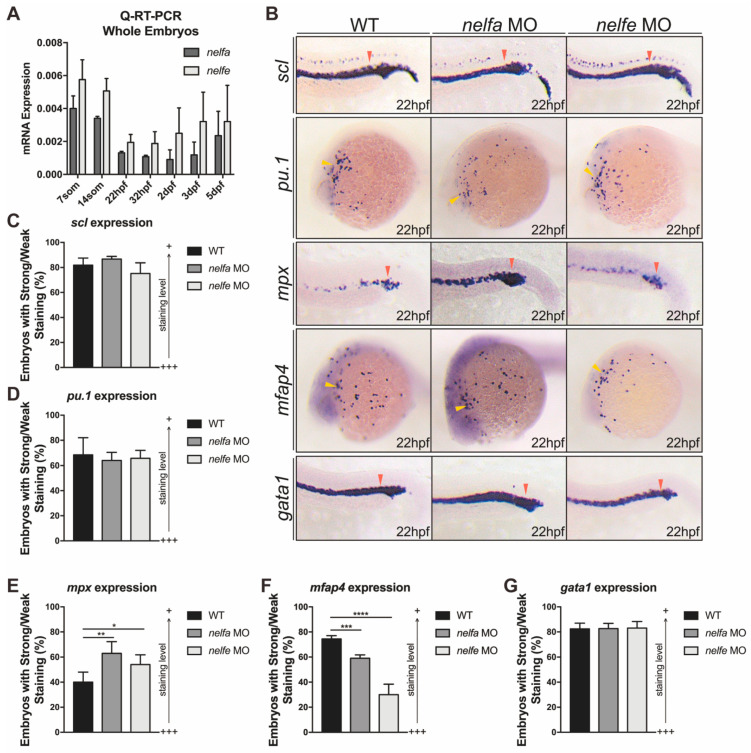
Other NELF subunits play similar roles in primitive granulocytic development. (**A**) Q-RT-PCR analysis of *nelfa* and *nelfe* expression at different stages. The relative gene expression was normalized to *β*-actin. (**B**) WISH for *scl*, *pu.1*, *mpx*, *mfap4*, or *gata1* in WT, *nelfa*, or *nelfe* morphants at 22 hpf. Yellow arrowheads and red arrowheads, respectively, indicate ALPM and PLPM. (**C**–**G**) Quantification of WISH results in (**B**) (*n* = 30–50 embryos per group). All results are presented as the mean ± SD from three independent experiments *t* test, * for *p* < 0.05, ** for *p* < 0.01, *** for *p* < 0.001, **** for *p* < 0.0001). “+++” and “+” in (**C**–**G**) respectively represent strong staining and weak staining.

NELF is a complex containing 4 subunits (NELFA, NELFB, NELFC/D, and NELFE) [12]. Only one copy of each NELF subunits is found in zebrafish, and all NELF subunits are highly conserved across different species. The individual zebrafish NELF subunit displays a more than 70% identity to the corresponding human homologous protein. We analyzed whether Nelfa and Nelfe play similar functions in primitive hematopoiesis. We first examined the expression of *nelfa* and *nelfe* at different development stages. *nelfa* and *nelfe* showed decreased mRNA expression as the embryos progressed, similar to the *nelfb* expression dynamics (Figure 5A). The results are also consistent with previous studies suggesting that the expression of NELF subunits is interdependent [28].

Next, we used *nelfa* and *nelfe* morpholinos as previously used [33] to respectively knock down their expression in the embryos (Supplementary Figure S2). Although morphants showed no significant difference in primitive progenitor and precursor development, indicated by *scl* and *pu.1* expression, excessive neutrophil differentiation in *nelfa* and *nelfe* morphants was observed, as suggested by increased *mpx* expression (Figure 5B–E). In accordance with the primitive macrophage development in *nelfb*^−/−^ mutants, *nelfa* and *nelfe* morphants showed decreased *mfap4* expression (Figure 5B,F). Primitive erythroid development was not affected, as indicated by the comparable expression of *gata1* with WT (Figure 5B,G). As a whole, the results suggested that other NELF subunits function similarly to Nelfb, and they may work together to specifically regulate granulopoiesis.

### 2.6. Granulocytic Differentiation at Late Developmental Stages and Adulthood Also Shows Defects in nelfb^−/−^ Zebrafish

**Figure 6:**
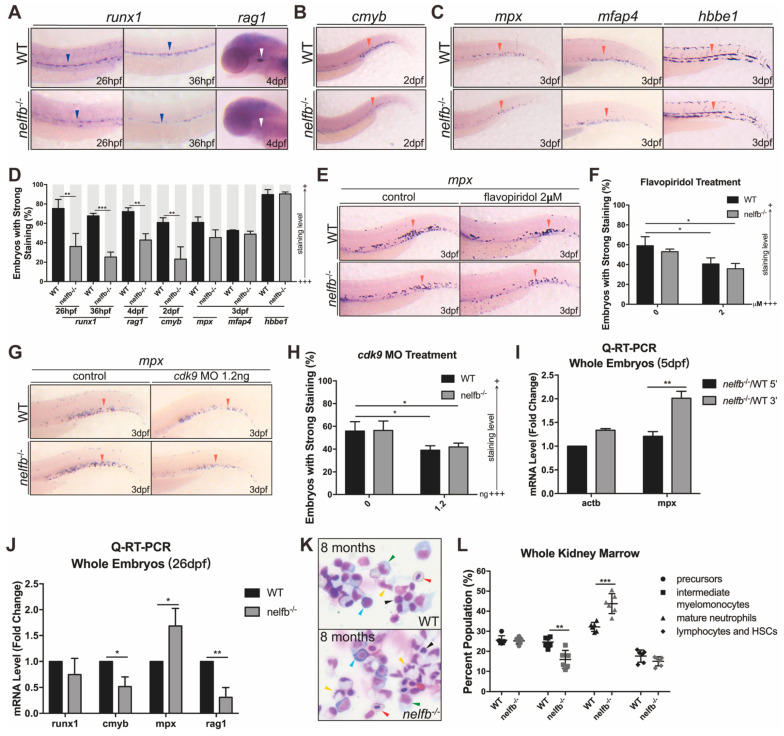
Granulocytic differentiation at late developmental stages and in adulthood also shows defects in *nelfb*^−/−^ zebrafish. (**A**) WISH for *runx1* or *rag1* in WT and *nelfb*^−/−^ embryos at 26 hpf and 4 dpf. (**B**) WISH for *cmyb* in WT and *nelfb*^−/−^ embryos at 2 dpf. (**C**) WISH for *mpx*, *mfap4*, and *hbbe1* in WT and *nelfb*^−/−^ embryos at 3 dpf. Blue arrowheads, white arrowheads, and red arrowheads, respectively, indicate AGM region, thymus, and CHT. (**D**) Quantification of WISH results in (**A**–**C**) (*n* = 30–40 embryos per group). (**E**) WISH for *mpx* at 3 dpf in WT and *nelfb*^−/−^ embryos treated with flavopiridol. (**F**) Quantification of WISH results in (**E**) (*n* = 20–30 embryos per group). (**G**) WISH for *mpx* at 3 dpf in WT and *nelfb*^−/−^ embryos injected with 1.2 ng *cdk9* MO. (**H**) Quantification of WISH results in (**H**) (*n* = 20–30 embryos per group). (**I**) Q-RT-PCR analysis of 5′ and 3′ transcripts of *mpx* gene in WT and *nelfb*^−/−^ embryos at 5 dpf. Gene expression is normalized to the 5′ transcript of *β*-actin and shown as fold-change relative to WT. (**J**) Q-RT-PCR analysis of hematopoiesis-related genes in WT and *nelfb*^−/−^ mutant fish at 26 dpf. Gene expression is normalized to *β*-actin and presented as fold-change relative to WT. (**K**) May–Grünwald–Giemsa staining of WKM from WT and *nelfb*^−/−^ fish at 8 mpf. Blue arrowheads, precursors; green arrowheads, intermediate myelomonocytes; yellow arrowheads, mature neutrophils; black arrowheads, lymphocytes and HSCs; red arrowheads, erythrocytes. (**L**) Quantification of WKM staining results. All results are presented as the mean ± SD from a representative of three independent experiments (*t* test, * for *p* < 0.05, ** for *p* < 0.01, *** for *p* < 0.001). “+++” and “+” in (**D**,**F**,**H**) respectively represent strong staining and weak staining. Note: dpf, days post-fertilization; mpf, months post-fertilization; AGM region, aorta-gonad-mesonephros region; CHT, caudal hematopoietic tissue; WKM, whole kidney marrow; HSCs, hematopoietic stem cells.

Next, we want to examine if *nelfb* also regulates granulopoiesis at later development stages and during adulthood. We analyzed the expression of hematopoietic genes in WT and *nelfb*^−/−^ mutants. Previous studies have shown that the disruption of Pol II pausing causes a reduction of hematopoietic stem cell (HSC) specification [33]. Our WISH results also revealed a loss of HSCs (*runx1* stained cells) from 26 hpf in *nelfb*^−/−^, which became more severe at 36 hpf. Lymphoid progenitors indicated by *rag1* that were closely related to HSC development also showed a dramatical decrease at 4 dpf (Figure 6A). The *cmyb*-stained definitive myeloid progenitors were also obviously reduced at 2 dpf (Figure 6B). However, the expression of *mpx*, *mfap4*, and *hbbe1* at 3 dpf showed no significant difference between the mutants and their WT siblings (Figure 6C). To test if transcription elongation regulates granulopoiesis during these later stages, we treated the embryos with flavopiridol and *cdk9* morpholino to prevent elongation. Consistent with the hematopoietic phenotypes during the early embryogenesis, the flavopiridol-treated embryos or *cdk9* morphants, with either WT or *nelfb*^−/−^ mutant backgrounds, showed increased HSCs and reduced neutrophils, indicating that granulocytic differentiation is similarly regulated during this stage (Supplementary Figures S3 and S6E–H). At 5 dpf, although *mpx* expression was not yet significantly increased in *nelfb*^−/−^, our studies show that the loss of *nelfb* led to a strongly increased release of Pol II at the *mpx* gene loci compared with the control *actin* loci, as demonstrated by the Q-RT-PCR analysis, which demonstrated that the 3′ end of the *mpx* gene transcripts displayed a higher expression level than the 5′ end gene transcripts (Figure 6I). These results suggested that granulocytic differentiation is similarly regulated by transcription proximal pausing and elongation during later developmental stages.

To further investigate myelopoiesis along the course of development, we tested the hematopoietic genes expression in 26-day-old zebrafish using Q-RT-PCR. The expression level of *runx1, cmyb*, and *rag1* remained slightly or significantly decreased in *nelfb*^−/−^. However, the *mpx* expression was strongly increased, supporting the idea that granulopoiesis is likely increased in *nelfb*^−/−^ as the development progresses, and that granulopoiesis is also enhanced during definitive hematopoiesis in *nelfb*^−/−^ (Figure 6J).

To study the hematopoietic phenotypes in adult zebrafish, a cytological assay of the whole kidney marrow (WKM) was performed. The overall kidney morphology between WT and *nelfb*^−/−^ were similar (Figure 6K). For the hematopoietic cells, the population of lymphoid/stem cells and the myeloid progenitor cells was slightly decreased. The intermediate myelomonocytes were significantly decreased, but the population of mature neutrophils was strongly expanded in *nelfb*^−/−^ (Figure 6L). These results implied that the loss of *nelfb* also induces the pre-maturation of granulocytic cells in adults.

Taken together, our findings support that Nelfb plays a consistent role in primitive and definitive granulocytic differentiation, likely through the regulation of Pol II pausing at the granulocyte lineage genes.

## 3. Discussion

Hematopoiesis is a very dynamic process, and multiple signals cooperate to control cell proliferation and differentiation [35]. To achieve the normal function of the hematopoietic system, the specific cell types must be generated at the correct time and place. The molecular circuitries underlying blood cell fate choice must enable cells to respond appropriately to the external stimuli [36]. Hematopoietic gene expression has been extensively studied at the transcription initiation step that is controlled by cell-specific transcription factors including SCL, GATA1, and PU.1 [37]. Recently, Pol II promoter proximal pausing has been reported as another critical step in various gene regulation [17]. This suggests that Pol II pausing allows for the integration of signals, but also maintains an active chromatin architecture, thus playing an essential role in a rapid and synchronized activation of gene expression on developmental or environmental changes [38,39], but whether Pol II pausing regulates hematopoietic lineage differentiation remains largely unknown. Previous in vitro studies suggested a role of NELF in hematopoietic differentiation [17]. Due to the embryonic lethality of NELF subunits (like NELFB) in knockout mice, the function of NELF during hematopoiesis remains uncharacterized [40]. Here, using the zebrafish model, we provide in vivo evidence that the NELF complex, an important promoter-proximal pausing factor, plays an essential role in regulating proper granulocytic development.

Interestingly, NELF-mediated pausing displays a lineage-specific regulation. It was previously shown that NELF protein levels remained unchanged during erythropoiesis [17]. Consistent with this study, our data showed that the loss of *nelfb* did not affect erythroid development, despite aberrant granulocyte formation. In humans, the half-life of circulating neutrophils is less than 1 day, while erythrocytes have an average life span of 120 days [41,42]. Moreover, as a crucial element of the immune system, granulocytes must be sensitive to environmental stress, capable of rapidly responding to multiple microbial and sterile challenges [42]. Granulopoiesis is a complex process that requires both lineage-specific transcription factors (such as PU.1 and C/EBPa) and ubiquitous transcription factors (such as STATs) [43]. Despite intrinsic factors, the development of granulocytic cells is also regulated by extrinsic factors such as cytokines and metabolic signaling [44,45]. The cooperation of these factors is the essential driving force in mediating granulopoiesis. Previous studies suggested that pausing is particularly prevalent and required in genes that are involved in stimulus-responsive networks [39]. Thus, we hypothesize that NELF-mediated Pol II pausing at granulocyte specific genes might initially suppress their expression, but then poise them to respond rapidly to appropriate environmental cues under steady-state and emergency conditions. It will be interesting to investigate the expression of NELF proteins in different lineage cells during hematopoiesis and to analyze the chromatin binding targets of NELF together with Pol II and hematopoietic lineage-specific transcription factors. Such studies will help reveal the precise mechanism of NELF in more dynamic cell-like granulocytes during cell differentiation. Moreover, the characterization of the role of NELF-mediated pausing during granulopoiesis under environmental stress, such as in cancer and injury, may also provide valuable information.

Our experiments prove that NELF plays a negative role in granulocytic development in vivo. Previous in vitro studies also showed that the forced expression of NELF subunits repressed granulocytic differentiation and enhanced hematopoietic progenitor cells [17]. Our data showed that the overexpression of human NELFB in blood cells led to a slight increase in hematopoietic progenitor cells, but did not interfere with normal granulocyte formation. It is possible that human NELFB may not express or function efficiently in zebrafish, despite the 77% identify between the two proteins. Nonetheless, it is possible that high expression of NELF may interfere with normal myeloid differentiation, leading to immature myeloid cells proliferation and accumulation, which is the main feature of myeloid leukemia. Interestingly, acute myeloid leukemia (AML) patients with both high NELFA or NELFB expression display low survival rates (Supplementary Figure S4). Thus, we provide evidence that inhibiting pausing or promoting elongation might be a new potential therapy for promoting myeloid differentiation in leukemia.

The promoter proximal pausing complex contains NELF and DSIF, which likely function together [6]. Previous studies have shown that the loss of DSIF pausing function had no effects on primitive hematopoiesis in zebrafish [33]. In our studies, the loss of *nelfb* led to pre-released Pol II elongation, resulting in decreased hematopoietic progenitor cells and excessive granulocytic development during primitive hematopoiesis. Thus, our studies suggest that there might be different pausing subtypes to control gene expression at different stages of development. Further studies focused on DSIF function during different stages, including definitive hematopoiesis, may help unveil more details about the differences between NELF and DSIF in regulating lineage specification.

In summary, we report that NELF-mediated transcription promoter proximal pausing plays an important role in preventing the premature development of granulocytic cells. It provides an in vivo model for future mechanistic studies on related processes and for understanding human hematopoietic diseases associated with dysregulated transcription pausing and elongation.

## 4. Materials and Methods

### 4.1. Zebrafish Maintenance and Embryo Handling

The wild type (WT) AB and transgenic zebrafish were maintained, handled, and bred according to standard protocols from the Institutional Animal Care and Use Committee of Shanghai Jiao Tong University. Adult zebrafish were raised in a circulating water system under a 14 h/10 h light/dark cycle at 26–28 °C and fed two times per day. Adult male and female zebrafish were kept separated using a transparent barrier in the same mating tank in the evening and mated the following morning. The embryos were collected and kept at 28.5 °C in E3 medium (5 mM NaCl, 0.17 mM KCl, 0.33 mM CaCl_2_, and 0.33 mM MgSO_4_) with a density of 80–120 embryos per 10 cm diameter Petri dish. Embryos were staged by hours post-fertilization (hpf) and days post-fertilization (dpf).

### 4.2. Generation of nelfb Knockout Mutants

*nelfb* knockout mutants were generated through the CRISPR-Cas9 system. *nelfb* specific guided RNA (gRNA) (Figure 1C) was designed to target on the exon 2. One-cell stage WT embryos were injected with 1 nL of the solution containing 100 ng/µL Cas9 mRNA and 20 ng/µL gRNA. Injected F0 fish were raised to adulthood and outcrossed with WT fish. F1 mutant offspring were identified using a T7 endonuclease I (T7E1) assay (M0302S, NEB, Bellingham, WA, USA) using primers around the target loci. Target loci was amplified by PCR from the genomic DNA, and the mutation was detected by Sanger DNA sequencing. F1 fish were outcrossed with WT to obtain stable F2 mutant lines. Primers for genotyping are listed in Supplementary Table S1.

### 4.3. Generation of NELFB Overexpression Lines

To overexpress NELFB in the blood system, the plasmid driven under the *drl* promoter, which contains the full-length of the human *NELFB* sequence, the zebrafish GFP sequence, and Tol2 mRNA, were co-injected into WT embryos at the one-cell stage. Injected embryos showing a positive expression of GFP were selected and raised to adults (F0 founder). F0 fish was outcrossed to WT fish, and GFP positive embryos was raised to adults (F1). F1 fish was outcrossed to WT again to obtain the stable transgenic line.

### 4.4. Whole-Mount In Situ Hybridization (WISH)

Whole-mount in situ hybridization was performed using digoxigenin-UTP labeled RNA probes (*scl*; *pu.1*; *runx1*; *cmyb*; *mpx*; *rag1*; *mfap4; gata1*; *hbbe1*). Embryos at the desired time point were fixed overnight in 4% paraformaldehyde (PFA) at 4 °C, bleached, and dehydrated in methanol at −20 °C for at least two hours. Further processing of the embryos was conducted according to the previous protocol [46]. The stained embryos were imaged with a SZX16 stereomicroscope (Olympus, Tokyo, Japan).

### 4.5. May–Grünwald–Giemsa Staining of Embryo Blood Cells and Adult Whole Kidney Marrow Cells

Twenty-eight hpf embryos were dechorionated with pronase (11459643001, Roche, Mannheim, Germany) and anesthetized in PBS containing 10% FBS and 0.02% tricaine. After tail clipping with a syringe needle, blood cells were collected by pipetting and cytospun onto slides by centrifugation at 450× *g* rpm for 3 min using Cytospin 4 (Sigma-Aldrich, St. Louis, MI, USA). Each group was collected from a pool of 80~100 embryos.

The adult fish kidney marrow was dissected and placed into PBS containing 10% FBS. The single hematopoietic cells from the kidney marrow were generated by pipetting and filtration through 40-µm filters. Single-cell suspension was diluted to 15,000–30,000 cells/mL and cytocentrifuged at 300× *g* rpm for 4 min using the Cytospin 4.

Blood smears were processed using May–Grünwald–Giemsa double stain (63590/48900, Sigma-Aldrich, St. Louis, MI, USA) for morphological analysis and differential cell counts.

### 4.6. Flow Cytometry and Cell Sorting

We sorted *drl*^+^ cells from Tg (*drl:GFP*) WT and from Tg (*drl:GFP*) *nelfb*^−/−^ embryos at 22 hpf. The embryos were dechorionated with pronase and washed with E3 solution. Single cells were collected by shredding the larvae with a blade, and then the cells were incubated for 20 min (37 °C) with 38 μg/mL Liberase (05401119001, Roche, Basel, Switzerland). A total of 10% FBS was added to stop the reaction, followed by filtration (40 µm filter) and centrifugation (1200× *g* rpm, 4 °C, 5 min). The supernatant was removed, and the cells were resuspended with PBS containing 1% FBS. GFP negative and positive cells were sorted respectively using FACS Aria III (Becton, Dickinson and Company, San Jose, CA, USA).

### 4.7. Gene Expression Tested by Real-Time qPCR

Gene expression was evaluated using real-time qPCR. Briefly, the total RNA was extracted from embryos with TRIzol reagent (10296028, Thermo Fisher Scientific, Waltham, MA, USA). The cDNAs were synthesized from the total RNA using the Hifair^®^ II 1st Strand cDNA Synthesis Super Mix (11123ES60, Yeasen, Shanghai, China). Hieff^®^ qPCR SYBR Green Master Mix (11203ES08, Yeasen, Shanghai, China) was used for qPCR analysis. Each target gene was calculated using the 2^−ΔΔCT^ method [47]. The primers for different target genes and β-actin (the reference gene) are listed in Supplementary Table S2.

### 4.8. Flavopiridol Treatment

The 2-somite embryos were continuously exposed to flavopiridol (146426-40-6, Aladdin, Shanghai, China) until 22 hpf. For later-stage treatment, 5-somite embryos were continuously exposed to flavopiridol until 36 hpf or until 3 dpf. The control groups were treated with DMSO. The embryos were fixed in 4%PFA and used for WISH.

### 4.9. Morpholino Microinjection

Morpholinos (gene tools) were injected into 1- to 2-cell-stage embryos. See Supplementary Table S3 for morpholino information. The efficiency of each morpholino was verified (Supplementary Figure S2).

### 4.10. Statistical Analysis

GraphPad Prism 7.0 software (GraphPad Software, San Diego, CA, USA, https://www.graphpad.com, accessed on 2 April 2016) was used to analyze all data. The values of all triplicate experiments are presented as mean ± SD. The statistical significance was displayed as “ns” for no statistical significance, “*” for *p* < 0.05, “**” for *p* < 0.01, “***” for *p* < 0.001, and “****” for *p* < 0.0001. The unpaired 2-tailed Student’s *t*-test was used for data analysis. For statistical analysis with WISH and other staining results, the groups with strong staining are used in the *t*-tests.

## Data Availability

The data presented in this study are available in article and Supplementary Materials.

## Supplementary Materials

The following are available online at https://www.mdpi.com/article/10.3390/ijms23073833/s1. References [33,48,49] are cited in the supplementary materials.
