# Supplementary material for: Negative Elongation Factor (NELF) Inhibits Premature Granulocytic Development in Zebrafish
Source: Int J Mol Sci. 2022 Mar 30;23(7):3833. doi: 10.3390/ijms23073833 (PMC8998717; doi:10.3390/ijms23073833)
Supplement: Supplementary file 1 [file ijms-23-03833-s001.zip › ijms-1648072-Supplementary Material.pdf]

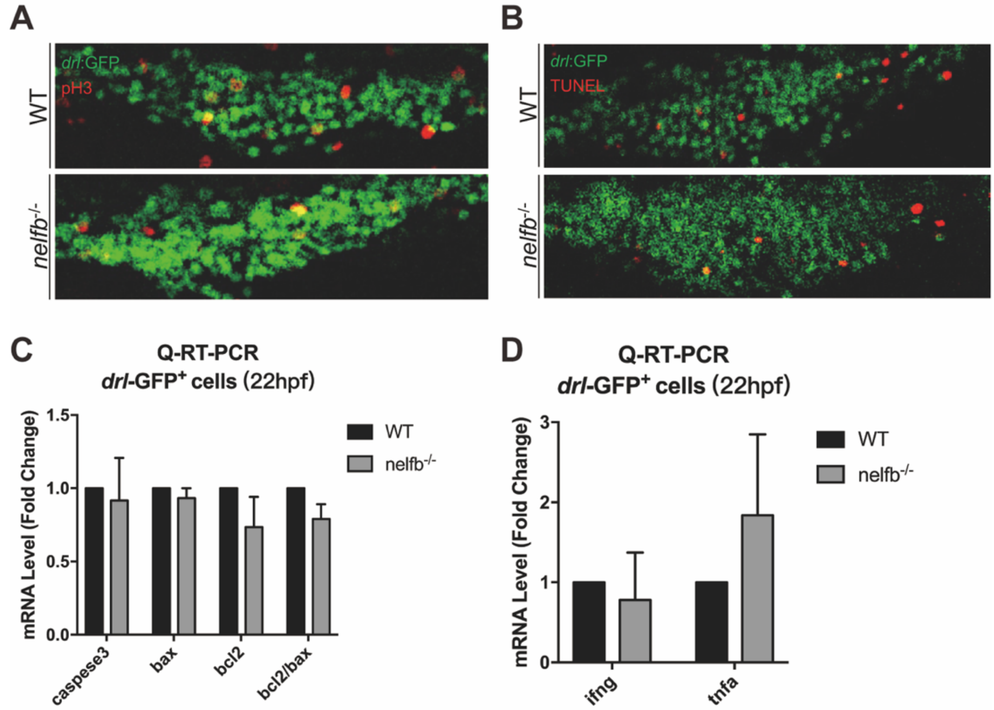

Supplementary Figure S1. Proliferation, apoptosis and inflammatory cytokine expression of hematopoietic cells in *nelfb*$^{-/-}$ mutants are normal compared with WT siblings.
(A) Double immunofluorescent staining for GFP (green) and phosphorylated histone H3 (pH3, red) in Tg(*drl:GFP*) embryos. **(B)** TUNEL staining in PBI of Tg(*drl:GFP*) embryos. **(C)** Q-RT-PCR analysis of apoptosis-related gene expression in sorted *drl-GFP*$^{+}$ cells in *nelfb*$^{-/-}$ and WT embryos at 22 hpf. **(D)** Q-RT-PCR analysis of inflammatory gene expression in sorted drl-GFP^{+} cells in *nelfb*$^{-/-}$ and WT embryos at 22 hpf. PBI, posterior blood island.

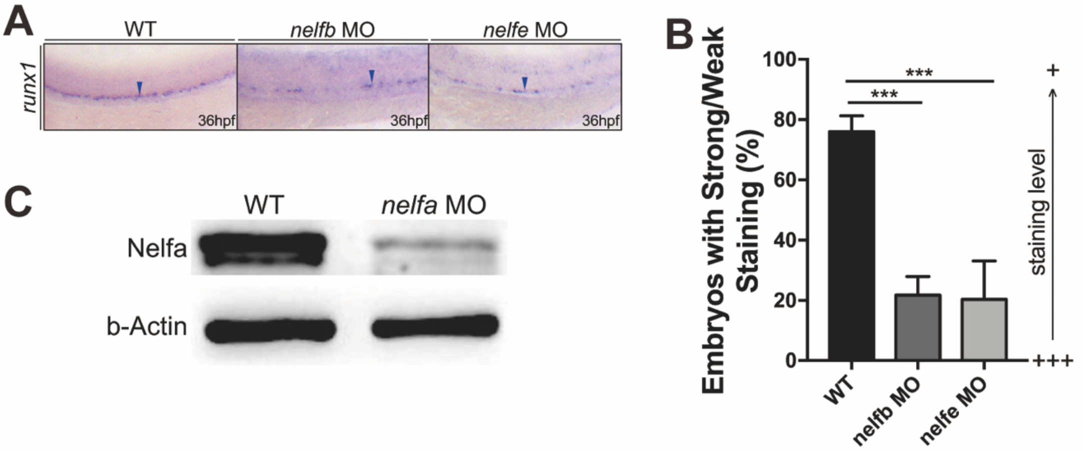

Supplementary Figure S2. *nelfa*, *nelfb* and *nelfe* mopholino knock-down efficiency.
(A) WISH for *runx1* in WT, *nelfb* or *nelfe* morphants at 36 hpf. **(B)** Quantification of WISH results in A (30-50 embryos per group). All results are presented as the mean ± SD from three independent experiments (t test, *** for p<0.001). **(C)** Western blot of Nelfa from WT and *nelfa* morphants at 22 hpf. B-Actin serves as a loading control.

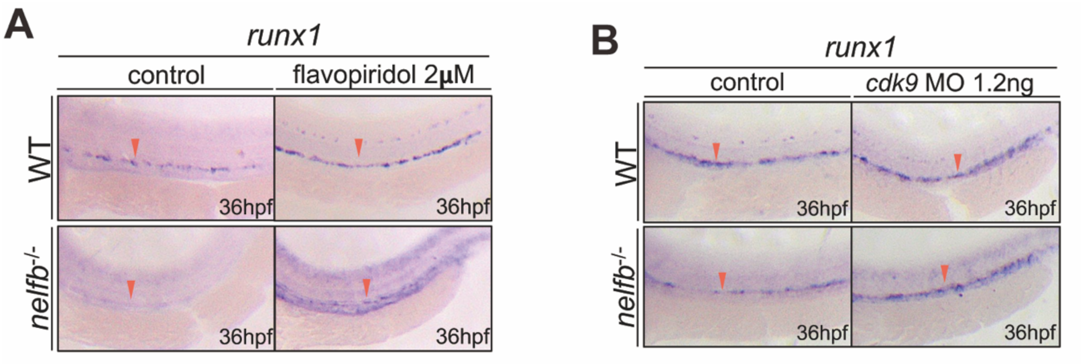

Supplementary Figure S3. CDK9 inhibition promotes HSC development.

(A, B) WISH for *runx1* at 36 hpf in wildtype and *nelfb*$^{-/-}$ embryos treated with flavopiridol or injected with 1.2 ng of *cdk9* MO, respectively. Orange arrowheads indicate AGM region.

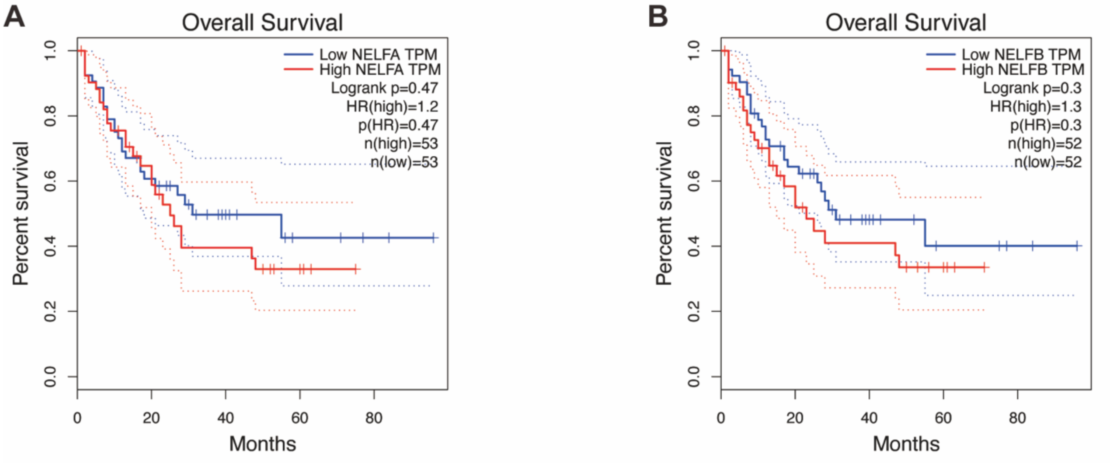

Supplementary Figure S4. Low NELF expression elevate AML patients' overall survival rate

(A, B) Overall survival (OS) analysis based on low- or high-expression of NELFA **(A)** or NELFB **(B)** by GEPIA (http://gepia.cancer-pku.cn) [1]

Supplementary Table S1. Primers for *nelfb*$^{-/-}$ genotyping

primer	sequence (5'→3')
sense primer	ACAGACGGAAAATGGCATATTG
antisense primer	TTCACCTGTCCTCCTCCTTG

Supplementary Table S2. Primers for Q-RT-PCR

gene	sense primer (5'→3')	antisense primer (5'→3')
actb (5' end)	gatcttcactccccttgttca	ataccggagccgttgtca
actb (3' end)	cctctcttgctccttccacc	actcctgcttgctgatccac
scl (5' end)	cggtgacctcggatgagaag	tcagcggaaattgctcggat
scl (3' end)	tcagcggaaattgctcggat	ttgctaggatacatcccatactgtt
pu.1 (5' end)	ccactgtcagggcaggttac	actcacgcaccttatcacgt
pu.1 (3' end)	caacaggaggagagcatggta	tgttccttttctcgatccac
mpx (5' end)	agcacaacttactctgagcttca	cagcccaccacaaaaagaaaagt
mpx (3' end)	tcgagccttggaaagagact	cctgctacaccctgtggac
mfap4 (5' end)	gaaccgagaactgctgtcct	gagtctcgtgctctgtgtca
mfap4 (3' end)	tatcggccgtggagagacta	tgaacttcttgtggcgtgtc
gata1 (5' end)	gataagcaagcaaacaggcg	agaggagttctccatctgca
gata1 (3' end)	tgagactttcgccacctga	cagtccacaagcattacagagg
runx1	cgtcttcacaaaccctcctcaa	gctttactgcttcatccggct
cmyb	tcgccagcttctaccaaa	cagggttgaggactttctgc
rag1	tgagagctgggaatgaacaca	ccgtgcggtacatcttgtga
nelfa	tggatcacgagaaaacccctg	aaacactgtgtccaccagcat
nelfb	aaggtgctgccgacttacat	tccttcaccgactcactgc
nelfe	gaatcagcagaccaggctgt	gagaagcccagacagatttcc

Supplementary Table S3. Mopholino Sequences

MO	sequence (5'→3')	reference
nelfa-ATG	CGCTCTCCCTCATCGACGCCATCTT	this article
nelfb-ATG	CCGGCGAACATTTTCACACGCTCTC	[2]
nelfe-ATG	AACTGGGAAATATTGCCATTTTGC	[2]
cdk9-spl	ACACACAAACATCAAATACTCACCC	[2]

Supplementary Methods

TUNEL immunostaining

The 22hpf Tg(*drl:GFP*) embryos were fixed in 4% PFA overnight at 4°C and dehydrated in 100% methanol at -20°C over 30 minutes. Further processing of embryos was conducted according to the previous article [3]. For analysis, the embryos were mounted in 1% low-melt agarose and imaged under a confocal microscope Leica SP8 microsystems (Leica, Heidelberg, DE, Germany).

Phosphorylated Histone 3 Immunostaining

The 22hpf Tg(*drl:GFP*) embryos were fixed in 4% PFA + 1% DMSO in 0.1M phosphate buffer (0.081M Na_2HPO_4, 0.014M NaH_2PO_4, pH7.4) overnight at 4°C. Embryos were dehydrated in 100% methanol at -20°C overnight after being washed with 0.1M phosphate buffer for 3 times. After digestion with pre-chilled acetone at -20°C for 15 minutes, the embryos were washed with incubation buffer (0.1M phosphate buffer + 0.2%BSA + 0.5%Triton-X) 15 minutes for 3 times and incubated with phosphorylated histone 3 primary antibody (#3377, CST) overnight at 4°C. The embryos were re-washed with incubation buffer 15 minutes for 3 times and incubated with coralite594-conjugated secondary antibody (SA00013-4, Proteintech) at room temperature for 4 hours.

The embryos were mounted in 1% low-melt agarose and imaged under a confocal microscope Leica SP8 microsystems (Leica, Heidelberg, DE, Germany) for analysis.

Westernblot

22 hpf embryos were dechorionated. Whole embryos were collected except yolk and treated with lysis buffer (RIPA lysis buffer (P0013B Beyotime Biotechnology, China) + 1mM PMSF (ST506 Beyotime Biotechnology, China)) on ice for 30 minutes to extract protein. Antibodies targeting b-Actin (66009-1, Proteintech, China) and NELFA (10456-1-AP, Proteintech, China) were used as the primary antibodies.

Reference

1. Wu, D., et al., *Pharmacological inhibition of dihydroorotate dehydrogenase induces apoptosis and differentiation in acute myeloid leukemia cells.* Haematologica, 2018. **103**(9): p. 1472-1483.
2. Yang, Q., et al., *RNA polymerase II pausing modulates hematopoietic stem cell emergence in zebrafish.* 2016. **128**(13): p. 1701.
3. Yu, S., et al., *BCAS2 is essential for hematopoietic stem and progenitor cell maintenance during zebrafish embryogenesis.* Blood, 2019. **133**(8): p. 805-815.
